# Cerina—Cognitive Behavioral Therapy–Based Mobile App for Managing Generalized Anxiety Disorder Symptoms Among University Students: Results From a Pilot Feasibility Randomized Controlled Trial

**DOI:** 10.2196/70691

**Published:** 2025-10-09

**Authors:** Ozlem Eylem-van Bergeijk, Tony Robinson, Matthew Manktelow, Michail Olympios, Siobhan Poulter, Prasannajeet Mane, Maria Panagioti, Joan Condell, Gerard Leavey

**Affiliations:** 1 Division of Population Health, Health Services Research and Primary Care University of Manchester Manchester United Kingdom; 2 Cerina Health London United Kingdom; 3 School of Computing, Engineering and Intelligent Systems Human Centred Computing, Intelligent Systems Research Centre Ulster University Londonderry United Kingdom; 4 Faculty of Medicine, School of Life Sciences Utrecht University Utrecht The Netherlands; 5 The Bamford Centre for Mental Health and Wellbeing, School of Psychology Ulster University Londonderry United Kingdom

**Keywords:** generalized anxiety disorder, mHealth, mobile health, CBT, students, feasibility RCT, randomized controlled trial, cognitive behavioral therapy

## Abstract

**Background:**

Generalized anxiety disorder (GAD) is common among university students due to academic pressure and financial uncertainty, among other challenges. Despite the need, the receipt of available psychological services is often low.

**Objective:**

This study investigates the feasibility of a digital unguided cognitive behavioral therapy (CBT)–based mobile app, Cerina, and examines the likely effects of this intervention in reducing GAD symptoms compared to the waitlist control group.

**Methods:**

Eligible students (n=158) with mild to moderate GAD symptoms were self-assessed through web-based questionnaires and were randomly allocated to the intervention group (n=79) or to the waitlist control group (n=79) following their informed consent. The intervention group had direct access to Cerina and followed CBT-based interactive sessions for 6 weeks. The waitlist control group participants had access to optional on-campus well-being services, and they were given access to Cerina 6 weeks after their randomization. Participants completed assessments on anxiety, depression, worry, and usability at three time points. Additionally, upon completing the intervention, they were invited to a web-based interview to understand the implementation of the intervention in more depth.

**Results:**

On average, 13% (10/79) intervention group participants dropped out, 61% (36/69) completed the core clinical content (2 sessions), and 12% (7/69) completed the desired number of sessions (6 or 7 sessions). Analyses of the completers (2 or more sessions) revealed significant group differences in GAD (mean 8.4, SD 3.7; *t*_42_=–2.25; *P*=.03; *d*=–0.7) and worry symptoms (mean 42.3, SD 10.8; *t*_42_=–2.50; *P*=.02; *d*=–0.8), as well as functional impairment (mean 16.7, SD 2.44; *t*_42_=–2.12; *P*=.04; *d*=–0.6) in favor of the intervention group at posttest with medium to large effect sizes. The intention-to-treat analyses confirmed significant group differences in GAD (mean 8.47, SD 2.7; *t*_156_=–2.23; *P*=.03; *d*=–0.4), and there were marginally nonsignificant group differences in worry symptoms (mean 41.5, SD 8.40; *t*_156_=–1.94; *P*=.05; *d*=–0.3) in favor of the intervention group at posttest with medium effect sizes. These results suggest that the intervention had a meaningful impact on reducing GAD symptoms and a modest impact on reducing worry symptoms among participants.

**Conclusions:**

The Cerina app showed promising results in reducing GAD symptoms among students. This result supports findings from other randomized controlled trials showing that digital CBT-based interventions are effective and feasible for a wide range of age groups and populations experiencing GAD symptoms. The low number of participants completing the recommended number of sessions suggests a usability issue. To address this, the intervention could be refined through an iterative design process informed by user feedback, and the long-term impact of specific engagement features in improving usability and retention could be assessed through extended evaluations.

**Trial Registration:**

ClinicalTrials.gov NCT06146530; https://clinicaltrials.gov/study/NCT06146530

**International Registered Report Identifier (IRRID):**

RR2-10.1136/bmjopen-2023-083554

## Introduction

### Background

Generalized anxiety disorder (GAD) is a common mental disorder (CMD) and is the most common anxiety disorder, affecting almost 30%-40% of the global population [1]. It is estimated that the prevalence of GAD varies widely across countries, with lifetime prevalence highest in high-income countries (5% [0.1%]), lower in middle-income countries (2.8% [0.1%]), and lowest in low-income countries (1.6% [0.1%]) [1,2]. Lifetime comorbidity is also high (81.9% [0.7%]), particularly with mood (63% [0.9%]) and other anxiety (51.7% [0.9%]) disorders [1-3]. Treatment is sought by approximately half of the affected individuals (49.2% [1.2%]) in high-income countries [1-3]. This suggests that GAD often goes unrecognized and untreated, even in high-income countries where access to mental health services is more readily available [4].

University students represent a vulnerable group at increased risk for anxiety disorders, particularly GAD. [5-12]. Data from the World Health Organization (WHO) World Mental Health International College Student project, conducted across 19 universities in eight countries (Australia, Belgium, Germany, Mexico, Northern Ireland, South Africa, Spain, and the United States), indicate that GAD was the most prevalent anxiety disorder among university students (n=14,371), with a lifetime prevalence of 18.6% and a 12-month prevalence of 16.7% [12]. Although longitudinal studies indicate mixed results about the increased risk for anxiety disorders among university students in the long run [13-16], there is growing evidence pointing to the subgroups of students who might be more at risk for anxiety disorders such as those who report financial uncertainty, those with a history of other CMDs [13-15], and students from ethnic minorities and lesbian, gay, bisexual, transgender, and queer groups [17].

According to the diathesis-stress model, it could be argued that common stressors faced by university students, such as academic pressure and financial uncertainty, may interact with pre-existing vulnerabilities, including a history of CMDs, developmental factors such as identity exploration and engagement in risky behaviors, and stressors specific to the university environment. Together, these interactions may heighten susceptibility to GAD in this population [16]. Additionally, Minority Stress Theory suggests that students from lesbian, gay, bisexual, transgender, and queer groups and ethnic minority backgrounds may face additional stressors, including discrimination and reduced access to social capital, which may further elevate their risk of developing GAD [18].

Psychotherapies are recommended for treating GAD [19,20]. Recent network meta-analysis examining the short- and long-term effects of different psychotherapies in reducing GAD symptoms suggested that cognitive behavioral therapy (CBT) remained significantly effective in reducing GAD symptoms at 3 to 12 months after completion of the intervention (standardized mean difference –0.60, 95% CI –0.99 to –0.21) [19]. However, relaxation therapy and third-wave CBTs were associated with short-term effectiveness [19]. Although pharmacotherapies are also recommended, CBT has larger overall treatment effects and fewer side effects than pharmacotherapy in the long run (6-12 mo follow-up; *g*=0.34) [21]. Although CBT represents the first-line therapy for GAD [20], social (eg, perceived stigma), organizational (eg, waiting times and service availability), and financial barriers (eg, in health care contexts where one must pay for their treatments) can restrict its access [22,23].

In recent years, digital CBT, including CBT-based smartphone apps and web-based interventions, has been proposed as an adjunct to or alternative to face-to-face CBT to overcome the above-mentioned barriers [24]. Despite the assumed benefits of digital CBT, such as reduced costs, anonymity, and accessibility [25], the existing randomized controlled trials (RCTs) point to mixed results about the effectiveness of these interventions in reducing GAD symptoms among student populations [26-31]. These mixed results may be attributed to the heterogeneity of CBT techniques used across treatment protocols, as well as methodological limitations such as small sample sizes, high dropout rates, and a lack of long-term follow-up [26]. Furthermore, high dropout rates suggest that the engagement with digital CBT might still be a challenge, especially among student populations [24-31]. For instance, an open feasibility trial [32] indicated that only a relatively small portion of a sample of university students, 26.5% (31/117), downloaded one or more of the CBT-based apps tested in the study, and approximately 24% (28/117) participants used these apps only once [32]. The literature indicates several person- and intervention-related reasons for dropout from digital CBT-based interventions. The most common being lengthy sessions conflicting with students’ busy academic schedules, needs for different kinds of help, and absence of personal contact [33,34]. There is, therefore, a need to improve adherence to digital CBT-based interventions and test new approaches to treat GAD symptoms among students [33].

One way of mitigating low engagement is through encouragement strategies such as automatic notifications, follow-up via phone or email, or automated feedback on homework exercises [35]. However, these methods are not prescriptive, and sustained engagement could be achieved if the intended population coleads the development and implementation of the digital CBT-based interventions [36,37]. More recently, technology-driven engagement features such as customized notifications, reminders, avatars (ie, web-based therapists), and chatbots have shown promise in changing users’ attitudes and behaviors [38-40]. These features differ from standard app functionalities by leveraging artificial intelligence to initiate interactions with users about specific content or exercises. Given the heterogeneity in mental health and help-seeking profiles among student populations [41-43], such technology-driven engagement strategies might mitigate the issue of low engagement [43,44].

### Aims

Overall, GAD is prevalent among university students, yet access to effective, scalable interventions remains limited due to barriers such as stigma, service availability, and time constraints. To help address this unmet need, Cerina Health, a UK-based mental health start-up, has developed Cerina, a mobile app grounded in CBT principles for the treatment of GAD symptoms. The app incorporates technology-driven engagement features within its user interface (UI), including customized notifications, reminders, and a chatbot that initiates conversations with users around specific therapeutic content or exercises, following co-design principles [36,37]. This study, therefore, has two primary objectives: (1) to investigate the feasibility of Cerina among a university student population, focusing on adherence, usability, and its potential for scaling to a full trial; and (2) to test the preliminary effects of Cerina in reducing GAD symptoms compared to a waitlist control group in university students presenting with self-reported GAD symptoms.

## Methods

### Study Design and Settings

The study is a single-blind pilot feasibility RCT with two conditions: an intervention group and a waitlist control group. This design addresses the primary research question: Is the Cerina app feasible to implement in a university setting in terms of usability, adherence, and potential for future scale-up? It also allows for the examination of the preliminary effects of Cerina compared to a waitlist control condition. A waitlist control group was selected to ensure that participants, many of whom may not otherwise have access to psychological support, would still have the opportunity to complete the intervention following the study [36]. Participants in the intervention group received immediate access to the Cerina app for 6 weeks, while those in the waitlist control group had access to optional on-campus well-being services during that period. After completing the posttest assessments at 6 weeks, the waitlist group was then offered access to Cerina.

All participants were asked to fill in web-based self-report measures of GAD, depression, worry, and work and social adjustment at baseline (t0), at 3 weeks (t1), and at 6 weeks (t2). Additionally, intervention group participants completed the system usability questionnaire at t2, while the waitlist group participants completed it after they accessed Cerina at t2. The 3-week (t1) assessment was included to capture early cognitive and behavioral changes, which are often expected to emerge within the first few sessions of CBT. The 6-week (t2) point aligns with the full delivery of core CBT components, and was used to assess short-term outcomes and initial consolidation of change. Semistructured interviews were conducted with all participants to explore which elements of the intervention facilitated or hindered participants’ progress during the study and how the intervention can be optimized to increase its acceptability, relevance, and user-friendliness in real-life settings. Intervention group participants were invited to the semistructured interviews right after they completed their follow-up assessments at t2. Waitlist control group participants were invited to the interviews after they accessed Cerina and completed their 6-week follow-up assessments.

The study was conducted in partnership between Cerina Health and Ulster University as part of the North West Europe INTERREG IT4Anxiety project. The primary objective of the IT4Anxiety project is to support the development and implementation of innovative, start-up-led solutions aimed at reducing anxiety. This collaboration supported Cerina Health’s research capacity to design and implement the study in a university setting. To avoid potential bias or conflicts of interest, the Chief Executive Officer of Cerina Health (PM) was involved only during the conceptualization phase of the study. The study was managed by OEB in collaboration with a research team at Ulster University. Study participant recruitment took place at Ulster University campuses from April 2023 until April 2024. Ulster University has approximately 30,000 students enrolled in undergraduate, postgraduate, and e-learning programs across its four main campuses: Belfast, Coleraine, Londonderry Derry, and Jordanstown in Northern Ireland.

### Ethical Considerations

Ethical approval for the study has been granted by the Ulster University Research Ethics Committee (FCPSY-22-084). The study adhered to the rights of the participants. All participants provided written informed consent on enrollment, and each participant had the right to make an informed, voluntary decision to participate in this study. They had the right to withdraw from the trial at any stage or access their data without any consequences. Participants also had the right to access available services offered by the university Student Well-being team, their general practitioner (GP), and additional resources (eg, Inspire Wellbeing, Samaritans, Lifeline). All data were stored according to the Ulster University General Data Protection Regulation. To adhere to privacy and confidentiality, each participant was given a participation number, and their data were stored in a deidentified format in the Ulster University infrastructure by the research team. User name and password were used, and the password was encrypted. The participant’s name and other identifiers were replaced by a unique code. To reduce the risk of disclosure, personal identifiers were stored separately from the research data at the Bamford Centre, Ulster University storage by the Principal Investigator and were only accessible to the research team. There was a key document that linked their participation number to their real identity. This was kept at the Bamford Centre, Ulster University storage, and only the research team and the Student Well-being team were able to access this key document as part of the safety protocol [36]. In addition, monetary compensation was not offered to the participants.

### Participants

Participants were adult students attending Ulster University who had reported anxiety symptoms based on the Generalized Anxiety Disorder Scale-7 (GAD-7) questionnaire. Eligible participants were (1) 18 years of age and older, (2) reported mild to severe self-reported GAD symptoms (ie, those who scored between 5 and 19 on the GAD-7 questionnaire were accepted as eligible), (3) enrolled as a student at Ulster University (ie, having a valid student ID number), (4) fluent in English, (5) had smartphone (ie, Android device or iPhone) and (6) internet connection, and (7) they gave informed consent. The exclusion criteria were (1) reporting minimal anxiety symptoms (ie, defined by a score of 5 and below on the GAD-7), (2) scoring 19 and above on the GAD-7, (3) reporting suicidal thoughts (ie, based on their scores on the Patient Health Questionnaire-9 [PHQ-9]), (4) having recently started taking psychotropic medication (ie, within the last 6 wk), and (5) not giving informed consent. Receiving psychological treatment was not part of the exclusion criteria.

### Recruitment and Randomization

Information about the study was available on Cerina Health’s website and Ulster University Student Well-being team’s website, where potential participants could register by filling out a web-based registration form. Those who registered received an email containing an information sheet, consent form, and a link to the baseline questionnaire. Participants who were eligible (based on the baseline questionnaire) and provided consent received a follow-up email informing them of their group allocation. Participants in the intervention group received further instructions on how to download the Cerina app, and technical assistance was provided if required. Those allocated to the waitlist control group received an email confirming their group allocation and were informed about the optional support services available through the Student Well-being team while waiting to access Cerina. All the participants, regardless of group allocation, received weekly email reminders to follow their sessions and complete their assessments.

Several channels were used to recruit participants: (1) invitation emails were circulated within the student email lists (global emails authorized by the university’s Pro Vice Chancellor for Academic Quality and Student Experience office), and the study-related communications (ie, banners, social media posts, and posters) were posted on Cerina Health’s LinkedIn and Instagram accounts, the Ulster University Student Well-being Facebook page, and the Ulster University Instagram account; (2) on-campus drop-ins (ie, brief face-to-face interactions between researchers and students where leaflets or posters are handed out to students and students asked questions about the study); and (3) Ulster University Students Union were approached in person and through emails to circulate information about the study within their networks of students.

The randomization scheme was derived using a random allocation software by an independent person who was not involved in the study. Randomization took place in blocks of 6 and in a 1:1 ratio. Allocation was concealed from the researchers involved in the study. It was not possible to mask participants due to the waitlist control group and the nature of the intervention. All participants were informed of their assigned condition. The study protocol is described in more detail elsewhere [36]. The ClinicalTrials.gov ID assigned to this study was NCT06146530.

### Intervention and the Waitlist Control Condition

Cerina is an unguided CBT-based mobile app for GAD symptoms. It does not provide any professional support related to the therapeutic content of the intervention [36,45]. If participants experience any difficulty with a particular therapeutic technique on the app or a technical problem, they are advised to contact the Cerina team at support@cerina.co. If the users experience a distressing situation unrelated to Cerina, they are advised to contact their GP or a health care professional. In addition, there is a self-care page available on the app, including links to external resources for further help, such as the National Health Service helpline, mental health charities, support groups, and Samaritans [36].

Following National Institute for Clinical Excellence guidelines for treating GAD and Panic Disorder [20], Cerina is positioned as a STEP 2 intervention: this includes low-intensity psychological interventions, individual guided self-help, and psychoeducation. The sessions are based on the treatment protocol of the Roth and Pilling UCL Competency Framework [46]. Clinical features include evaluation of worry (using the Penn State Worry Questionnaire [PSWQ]), worry awareness training, understanding and coping with intolerance of uncertainty, re-evaluating beliefs about the usefulness of worrying, worry management techniques, problem-solving strategies, cognitive exposure, and relapse prevention [20,47]. Cerina consists of 7 sessions of CBT for the treatment of GAD. Each session includes information, tasks, and exercises to help the participants understand GAD, the CBT approach, and how it applies to them. The participants complete the sessions in a progressive direction, and they are advised to complete the first six sessions on a weekly basis, that is, one per week. However, they can repeat a session before going on to the next session. The last session is designed as a review of progress session rather than a full therapy session and does not include additional exercises or homework assignments. These seven therapy sessions are (1) learning about GAD, (2) dealing with worry, (3) managing worry and anxiety, (4) beliefs about worry, (5) managing uncertainty, (6) testing beliefs, and (7) review and therapy blueprint [36].

Several design features were identified during the co-design process [36] and were incorporated into the UI to promote user engagement. These include (1) vibrant and simple illustrations to present the case studies in each session; (2) customized notification settings; (3) a separate self-care page including podcasts, anxiety management techniques, and available resources for further help if needed; (4) regular check-ins to rate mood; (5) a safety mechanism embedded within the participants’ conversations with the chatbot, for instance, if a participant talks about death and suicidal thoughts, the chatbot provides contact details of the available crisis lines and services for further help; and (6) flexibility regarding the navigation, for example, participants can resume where they are left off upon logging into the app, and they can revisit previous sessions or review their therapy journey at any time [36].

The waitlist control condition includes optional on-campus well-being services offered by the Student Well-being team. The Student Well-being team on the Belfast, Coleraine, and Derry Londonderry campuses provides free and confidential support and guidance to students with a broad range of issues, concerns, and challenges, helping them to successfully engage in their studies. Student Well-being assistants provide an initial assessment to determine a student’s primary need, and then well-being advisers, student mental health advisers, and accessibility advisers are available to provide a variety of solution-focused interventions based on that need. Each campus team is led by a Student Well-being manager to support the advisers in the management of risk and response to clinical incidents. Additionally, therapeutic counseling support is also available free to students through the external counseling provider via a dedicated 24/7 counseling helpline.

### Study Outcomes

#### Primary Outcome

Feasibility was defined in terms of adherence to the intervention, its usability, and potential to deliver a full RCT in the future [47].

Adherence was defined based on Staudt adherence model [37,48], and it includes behavioral (ie, performance on tasks that are necessary to implement the intervention in daily life, such as homework completion) and attitudinal (ie, emotional commitment to the intervention) adherence [48]. In this study, behavioral adherence was assessed based on the number of (%) login attempts and the number of (%) participants who followed the sessions [30]. Attitudinal adherence was measured based on the number of participants (%) who completed the core therapeutic content (ie, entries in their worry diary in session 1 and reflections in session 2), some therapeutic content (ie, psychoeducational part), or no therapeutic content at all (ie, few taps and invalid entries) [36]. Additionally, we looked at the desired number of sessions followed (n=6) by the intervention and waitlist group participants.

Usability was measured with the System Usability Scale (SUS) [49]. The SUS consists of 10 items rated on a 5-point Likert scale, with total scores ranging from 0 to 100. It has demonstrated good reliability (Cronbach α=0.91) [49]. The scale also showed good internal consistency in this study (α=0.81). Scores of 70 and above are considered indicative of high usability, suggesting that the intervention is easy to use and navigate, and that users feel confident implementing it in real-life contexts [49]. Scores between 50 and 70 indicate acceptable usability, though they may point to minor issues or concerns in everyday use. Scores below 50 raise major concerns about usability and warrant further investigation [49]. SUS results should be interpreted alongside data from semistructured interviews to inform potential refinements to the intervention [49].

The potential to deliver a full-scale RCT in the future was assessed based on the response rates to the self-report questionnaires, the recruitment procedures, and participants’ willingness to be randomized to a waitlist control group [36].

#### Secondary Outcomes

GAD-7, PSWQ-PW, PHQ-9, and the Work and Social Adjustment Scale (WSAS) were selected secondary outcome measures as these instruments are routinely used to assess clinical symptoms across diverse settings [50-54].

GAD symptoms were assessed through the GAD-7 [50], a 7-item self-report scale designed to identify the severity of GAD. Scores range from 0 to 21, with a cutoff score of 5 used to distinguish between clinical and nonclinical populations. The scale has good psychometric properties [50]. In this study, the scale showed acceptable internal consistency (α=0.7).

Worry symptoms were assessed using the PSWQ-PW [50], a 15-item inventory assessing both the weekly status of pathological worry and treatment-related changes of worry during the treatment [50]. Each item is scored on a 7-point rating scale, ranging from 0=never to 6=almost always. The total score ranges from 0 to 90, with a high score indicating higher levels of worry. PSWQ-PW shows good reliability and convergent validity [50]. In this study, the scale showed good internal consistency (α=0.8).

Depression symptoms were assessed using the PHQ-9 instrument [51]. Scores range from 0 to 27, with a score of 10 and above considered to be a clinically significant level of depression. The PHQ-9 has good reliability and validity [51]. In this study, the scale showed good internal consistency (α=0.8).

Functional impairment was assessed using WSAS [52]. Scores range from 0 to 40. The scale assesses the impact on work, home, social and private activities, and personal and family relationships. A score of 20 and above is considered to indicate severe functional impairment, scores between 10 and 20 suggest severe but functional impairment, and scores of 10 and less are considered subclinical. The scale has good reliability and validity [52]. In this study, the scale showed good internal consistency (α=0.8).

### Statistical Analysis

The process evaluation examined usability (ie, pathways and barriers to using Cerina in daily life), the clinical utility of the app, the context in which the implementation of the clinical content takes place, and the processes involved in delivering the intervention [36]. These aspects were investigated through semistructured web-based interviews or web-based questionnaires with participants from both the intervention and waitlist control groups who completed the intervention. The results of the process evaluation are reported in detail elsewhere [55].

The RCT was conducted in accordance with the CONSORT (Consolidated Standards of Reporting Trials; Multimedia Appendix 1) and CONSORT-EHEALTH (Consolidated Standards of Reporting Trials of Electronic and Mobile Health Applications and Online Telehealth) [56] checklists (Multimedia Appendix 2). First, 2-tailed *t* tests and chi-square tests were performed using SPSS (IBM Corp) to compare the baseline characteristics between participants allocated to the intervention group and those allocated to the waitlist control group. Second, independent 2-tailed *t* tests were used to compare changes in secondary outcomes across time points between the two groups. These analyses were conducted in RStudio (version 3.6.1; Posit Software, PBC) using the completers dataset [57]. A 2-tailed *t* test was chosen instead of a linear mixed model with random intercepts, as it is equivalent in terms of yielding the same test statistic for evaluating the treatment effect [58]. Third, after reviewing the data collected, 41.83% of the dataset was missing; this missing data was deemed to be completely at random [59]. To address the missing data, a single imputation-linear regression model [60] was trained, using scikit-learn in Python (version 3.11.0; Python Software Foundation) to estimate and balance the missing data [61]. The model was trained per item, based on the pre-existing data of weeks t0, t1, and t2, with a “bias gradient” represented by the items’ values at t0. Therefore, a feature prediction was made for each (row) subject considering their t0 outcomes, the pre-existing t1 and t2 results, and the “bias gradient” created. The rationale for this imputation technique was three-fold: (1) it factors a multivariate approach, considering the relationship between the items [62,63], and the relationship and underlying patterns between the same item in a longitudinal nature [60]; (2) it illustrates a linearity between the three time points, showcasing a decrease of symptomatology based on the existed, preimputation data; and (3) it creates an acceptable normal distribution [64] in comparison with the pre-existed data with a “trade-off” of shifting the residuals, extreme values toward the mean (decreasing variance of residuals) [65]. Following this data analysis and transformation, the intention-to-treat (ITT) analyses were performed to test the likely effects of the intervention compared to the waitlist control group, as described for the completers dataset. Cohen *d* for the effect of the intervention was estimated by calculating the difference between estimated means (corrected for baseline) divided by the raw pooled SD. Effect sizes of 0.8 were accepted as large, effect sizes of 0.5 were moderate, and effect sizes of 0.2 were small [66]. A two-sided *P*<.05 indicated statistical significance.

## Results

### Participants

This subsection provides information on the recruitment channels, participant flow throughout the study, and baseline characteristics and their distribution across the intervention and the waitlist control group.

Overall, a total of 514 students registered for the study through the registration link provided in the global emails (n=488, 95%), social media channels including Instagram (n=3, 0.6%), Facebook (n=4, 0.7%), LinkedIn (n=1, 0.2%) and on the leaflets and posters (n=4, 0.7%) shared during the on-campus drop-ins. Additionally, a few participants registered for the study through word of mouth from the Student Well-being team (n=3, 0.6%), friend (n=2, 0.4%), and tutor (n=2, 0.4%).

**Figure 1:**
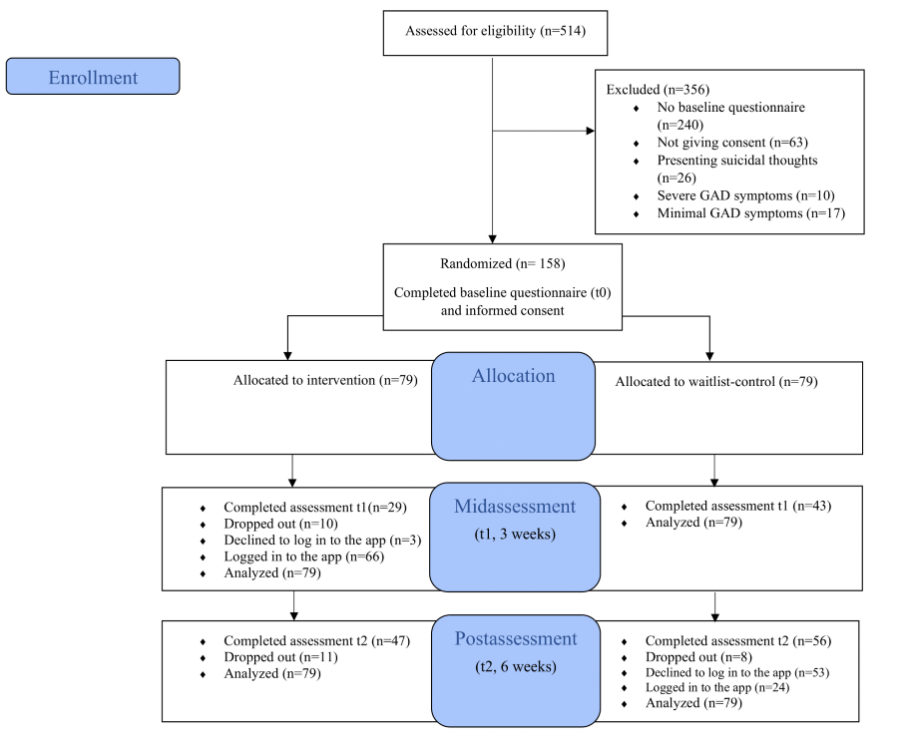
Participant flowchart. GAD: generalized anxiety disorder.

Figure 1 shows the participant flow through the trial. Out of 514 registered people, 65% (n=356) were excluded mainly due to not completing the baseline questionnaire (n=240, 47%), not giving consent (n=63, 12%), presenting suicidal thoughts (n=26, 5%), presenting severe GAD symptoms (n=10, 2%), and presenting minimal GAD symptoms (n=17, 3%).

The remaining 158 people were eligible, gave informed consent, and were randomized (n=79 intervention; n=79 waitlist control group). In the intervention group, a total of 37% (n=29) of participants completed their 3-week assessments, and a total of 59% (n=47) of participants completed their 6-week postassessments. In the waitlist control group, a total of 54% (n=43) of participants completed their 3-week assessments, and a total of 71% (n=56) of participants completed their 6-week assessments. Additionally, all participants who used Cerina and completed their 6-week assessments (n=56, 54%) were invited to the web-based postassessment feedback interviews. Of those who agreed to give postassessment feedback (n=20, 36%), the majority of them opted out of interviews and completed web-based questionnaires (n=16, 80%), while only 20% (n=4) agreed to be interviewed.

A significantly higher number of participants in the intervention group (n=41, 71%) than in the waitlist control group (n=17, 29%) did not complete their 3- and 6-week assessments (*P*<.05). There were no significant differences in baseline characteristics between participants who completed the assessments and those who did not.

Multimedia Appendix 3 displays the baseline characteristics for all the participants included in the study. Overall, there were no imbalances between the intervention and the waitlist group participants. The majority were female (n=129, 81.6%), undergraduate students (n=78, 49.4%), and aged between 18 and 30 years (n=104, 65.8%). While a considerable number of participants chose friends and family as their main source of informal support (n=42, 26.5%), the majority of them were not receiving professional support at the time of their baseline assessment (n=83, 52.5%). Of those who were receiving professional support, 13.3% (n=21) chose GP as the main source of professional support. More than half of the participants (n=105, 66.5%) considered professional support as neither helpful nor unhelpful. While the majority of participants were not using psychotropic medication for a mental health problem (n=105, 66.5%), a considerable number of them (n=53, 34%) were using psychotropic medication at the time of the baseline assessment. Of these, 24.7% (n=39) of participants had been on a stable dose for more than 6 months, 6.3% (n=10) for the past 2 months, and 2.5% (n=4) had recently started medication.

On average, participants reported moderate levels of generalized anxiety symptoms (mean 11.64, SD 3.84), clinically significant levels of depression (mean 12.71, SD 5.16), a substantial level of worry (mean 51.08, SD 8.75), and severe but functional levels of impairment (mean 19.41, SD 8.53). Finally, there were no statistically significant differences between the intervention and waitlist control groups on any baseline characteristics.

### Safety

This subsection summarizes information on the implementation of the safety protocol throughout the study.

As part of the safety protocol, students (n=26) who reported suicidal thoughts at the baseline assessment (measured by PHQ-9) were signposted to the Student Well-being team and excluded from the study. These student participants received a phone call from the Student Well-being team to schedule a follow-up phone call with an adviser for further assessment. Out of those who were signposted, 27% (n=7) did not respond, 38% (n=10) did not require support, 27% (n=7) started engaging with the Student Well-being team, and the remaining were receiving other external support (n=2, 8%). In addition, all participants were closely monitored throughout the study via their responses to regular in-app check-in questions and their 3- and 6-week assessments. None of the participants’ responses indicated worsening of symptoms, and none of the participants contacted the research team to be signposted to the Student Well-being team for further help.

### Feasibility

This subsection presents findings related to participants’ adherence to the intervention and the usability of the Cerina app.

#### Behavioral Adherence

Of the 79 participants allocated to the intervention group, 13% (n=10) dropped out, and 87% (n=69) were given access to Cerina. Among those, 97% (n=66) logged in to Cerina, and more than three-quarters (n=59, 89.3%) followed the sessions. In the waitlist control group, less than half (n=26, 33%) were given access to Cerina, while the remaining participants (n=53, 67%) did not follow the sessions. Of the 26 waitlist group participants who were given access to Cerina, the majority (n=24, 92.3%) logged in to the app, and 96% (n=23) followed the sessions.

#### Attitudinal Adherence

On average, 61% (n=36) of intervention group participants engaged with the core therapeutic content (ie, have entries in their worry diary in session 1 and reflect on their worry diary in session 2), while 25% (n=15) engaged with the psychoeducational content in session 1, and 14% (n=8) did not engage with any content (ie, few taps and invalid entries). Additionally, only 12% (n=7) of participants completed 6 or 7 sessions in the intervention group. Similarly, of all the waitlist group participants who followed the sessions, 56% (n=13) engaged with the core therapeutic content, 35% (n=8) engaged with psychoeducational content in session 1, and 9% (n=2) did not engage with any content. Only 4% (n=1) of participants completed 6 or 7 sessions in the waitlist control group.

#### Usability

Participants in the intervention group reported an average score of 71.08 (SD 12.04) at 6-week assessments (t2) on the SUS scale. This score indicates highly acceptable usability [49] and suggests that overall, the intervention was considered easy to use, various UI functions were well-integrated, and users felt confident while using it.

### Intervention Effects

This subsection presents the results of the completers and the ITT analyses.

#### Completers Analyses

Overall, the completers analyses indicated changes in secondary outcomes: GAD-7, PHQ-9, PSWQ-PW, and WSAS scores from baseline (t0) to 6-week (t2) assessments.

The results of the independent 2-tailed *t* test indicated that there was significantly greater reduction in GAD-7 scores among participants in the intervention group (mean 8.4, SD 3.7; 95% CI 6.66-9.86) compared to the waitlist control group (mean 11, SD 3.9; 95% CI 8.39-12.52; *t*_42_=–2.25; *P*=.03) at t2 with moderate to large effect size (*d*=–0.7), suggesting that the intervention had a meaningful impact on reducing GAD-7 scores among completers. The point bilateral correlation (*r*=–0.328) supports this finding (Multimedia Appendix 4).

There was significantly greater reduction in PSWQ-PW scores among participants in the intervention group (mean 42.3, SD 10.8; 95% CI 36.3-46.31) compared to the waitlist control group (mean 49.8, SD 9.06; 95% CI 43.86-52.81; *t*_42_=–2.50; *P*=.02) at t2 with large effect sizes (*d*=–0.8), suggesting that the intervention had a strong impact on reducing PSWQ-PW scores among completers. The point-biserial correlation (*r=*–0.360) indicates that the intervention may be highly effective in alleviating worry symptoms among completers (Multimedia Appendix 5).

Similarly, the independent 2-tailed *t* test results comparting the intervention (mean 16.7, SD 2.44; 95% CI 11.99-21.66) and the waitlist control group (mean 23.2, SD 8.35; 95% CI 16.38-25.12) on WSAS scores revealed significant group differences (*t*_42_=–2.12; *P*=.04) with moderate effect size (*d*=–0.6) at t2 in favor of the intervention, suggesting that the intervention had a meaningful impact on reducing WSAS scores among completers. The point bilateral correlation (*r*=–0.311) supports the notable impact of the intervention on improving completers’ functional impairment (Multimedia Appendix 6).

Finally, the independent 2-tailed *t* test results comparing the intervention (mean 10.1, SD 6.1; 95% CI 7.23-12.51) and the waitlist control group (mean 13.3, SD 7.1; 95% CI 9.06-15.53) on PHQ-9 scores at t2 did not reveal significant group differences (*t*=–1.5678; *P*=.12) with moderate effect size (*d*=–0.5) suggesting some potential impact of the intervention on depression symptoms among completers, and the point-biserial correlation (*r*=–0.2351) suggest a trend points toward the intervention having a beneficial effect among completers (Multimedia Appendix 7).

#### ITT Analyses

The overall changes in secondary outcomes from t0 to t2 were also observed in the ITT analyses (Table 1).

There was significantly greater reduction in GAD-7 scores among participants in the intervention group (mean 8.47, SD 2.7; 95% CI 7.86-9.07) compared to the waitlist control group (mean 9.6, SD 3.8; 95% CI 8.79-10.47; *t*_156_=–2.23; *P*=.03) at t2 with moderate effect size (*d*=–0.4), suggesting that the intervention had a meaningful impact on reducing GAD-7 scores. The point-biserial correlation (*r*=–0.176) supports the conclusion of a moderate effect (Multimedia Appendix 8).

There were marginally nonsignificant group differences between the intervention (mean 41.5, SD 8.40; 95% CI 36.65-43.41) and waitlist control group (mean 44.2, SD 8.9; 95% CI 42.21-46.22) in PSWQ-PW scores (*t*_156_=–1.94; *P*=.05) at t2 with moderate effect size (*d*=–0.3), suggesting that the intervention had a modest impact on reducing PSWQ-PW scores. The point-biserial correlation (*r*=–0.154) supports the conclusion of a moderate effect (Multimedia Appendix 9).

The independent 2-tailed *t* test results comparing the intervention and the waitlist control group on PHQ-9 and WSAS scores at t2 did not reveal significant group differences with small effect sizes (Multimedia Appendices 10 and 11).

**Table 1:** Mean changes from baseline to posttest (n=158).

Secondary outcomes	Intervention (n=79), mean (SD)		Waitlist control (n=79), mean (SD)			Cohen *d*
	Baseline (t0)	Posttest (t2)	Baseline (t0)	Posttest (t2)		
					
GAD-7^a^	12.25 (3.70)	8.47 (2.71)^b^	11.03 (3.89)	9.63 (3.76)	–0.355	
PHQ-9^c^	13.73 (5.16)	10.00 (3.99)	11.68 (4.99)	10.78 (5.40)	–0.165	
PSWQ^d^	50.96 (8.65)	41.53 (8.40)	51.19 (8.90)	44.21 (8.96)	–0.309	
WSAS^e^	20.94 (7.85)	17.21 (7.75)	17.89 (8.61)	18.25 (7.80)	–0.133	

^a^GAD-7: Generalized Anxiety Disorder Scale-7.

^b^*P*<.05.

^c^PHQ-9: Patient Health Questionnaire-9.

^d^PSWQ: Penn State Worry Questionnaire.

^e^WSAS: Work and Social Adjustment Scale.

#### Correlations Between the Support Measures and Secondary Outcomes

We investigated the correlations between the support measures (psychological support, professional support, and helpfulness of the professional support) at baseline (t0) and the improvements in the secondary outcomes to explore whether any of the support measures could have predicted the improvements in secondary outcomes at 6 weeks (t2; Multimedia Appendix 12).

The correlations between psychological support at t0 and the secondary outcomes were all negative, with the strongest negative correlation observed with WSAS scores (*r*=–0.186). This suggests that higher psychological support at t0 was associated with lower scores on secondary outcomes, indicating a reduction in anxiety, worry, depression, and functional impairment.

Overall, the correlations between professional support at t0 and the secondary outcomes were very weak. There is a slight positive correlation with PHQ-9 (*r*=0.058) and WSAS (*r*=0.126) scores. This implies that professional support at t0 had a minimal impact on secondary outcomes, indicating almost no relationship.

The correlations involving helpfulness of the professional support at t0 were also very weak, with the highest correlation being with WSAS (*r*=0.038) scores. This indicates that the perceived helpfulness of professional support at t0 had a negligible relationship with secondary outcomes.

## Discussion

### Principal Results

This pilot feasibility RCT included 158 Ulster University students, who at baseline reported moderate levels of generalized anxiety, depression, worry symptoms, and severe but functional levels of impairment. Students’ interest in registering for the study, along with the safety of the trial procedures, which did not restrict access to the existing Student Well-being services, provided supportive evidence for the feasibility of the current trial. These procedures, including risk monitoring and participant autonomy in accessing care, align with ethical standards for low-risk digital intervention trials and offer a model for future studies in similar populations.

Although many participants engaged with the Cerina app, a notable proportion did not complete the desired number of sessions and skipped their 3-week assessments (t1). Nevertheless, the ITT analyses revealed significant group differences in GAD symptoms and marginally nonsignificant group differences in worry symptoms in favor of the intervention group with medium effect sizes at 6-week assessments (t2). These medium effect sizes are consistent with benchmarks reported in digital CBT-based interventions, which typically range between 0.3 and 0.6 for anxiety symptoms [19,21,23,26-31]. This indicates that even with limited engagement, the Cerina app was able to produce clinically meaningful improvements in GAD symptoms. However, it is plausible that adherence challenges may have diluted the observed effects, particularly in the ITT analyses.

Future iterations of the app could consider enhancing personalization, integrating gamification, or incorporating brief synchronous contact to optimize engagement and maximize therapeutic benefits. The digital format, along with the successful student-led recruitment, indicates that this intervention could be scaled across university contexts, especially where institutional counseling services are overburdened. However, to broaden accessibility, future adaptations may need to consider linguistic, cultural, and digital literacy differences to ensure inclusivity in more diverse educational or community-based settings.

### Participants

Overall, the total number of students registered for the study (n=514) indicates university students’ interest in line with the existing literature on the reach and access of the mental health apps among this population [33,67].

Despite the anticipated potential of social network channels such as Facebook and Instagram to support the recruitment for digital interventions [68,69], the majority of the students in this study self-registered through the university’s global email system. It may be that different communication channels need to be adapted to the target population’s needs, enabling age- and culture-specific implementation strategies, as well as content adaptations to enhance relevance and effectiveness [35]. Furthermore, in the context of student populations, present results suggest that using recruitment channels that are of interest to students and using time-efficient strategies providing anonymity could be important during the recruitment process [33,67]. This highlights the effectiveness of digital, anonymous, and institution-based recruitment strategies in university settings. Compared to previous studies with limited uptake [32,47], our approach facilitated self-initiated participation with minimal burden on institutional services—an important strength for scalable interventions.

The baseline characteristics of the included participants in this study are consistent with findings from existing studies on digital CBT-based interventions [26-32] and the World Mental Health International College Student study [12,42,70]. Notably, in this study, the considerable number of students on medication because of mental health problems (n=53, 34%) provides further support for the prevalence of CMDs, their severity and complexity, particularly among first-year students transitioning from adolescence to adulthood [12]. Supporting this, data from a retrospective cohort study looking at the use of psychotropic medication among student populations between 2007 and 2019 in America reported a 2-fold increase in use of medication in nearly all classes of medication, including mood stabilizers and antianxiety medication [71]. In this study, 24.7% (n=39) of participants reported being on a stable dose of medication for the past 6 months. This is comparable to the number of students (n=65,899, 20.5%) who reported being prescribed medication because of mental health problems over the past 12 months [71].

The combination of high rates of psychotropic medication use and low uptake of psychological treatment among student populations in general [51] and in Northern Ireland specifically [12] underscores the potential of digital CBT-based interventions to improve access to first-line psychological help for GAD and other CMD symptoms among university students [72-77]. These findings are particularly relevant for universities with limited counseling capacity, as digital CBT offers a scalable adjunct or alternative pathway for mental health support.

### Safety

In alignment with ethical standards, the safety procedures in this study were integrated with Ulster University’s Student Well-being services, allowing participants to access the available psychological services throughout the study. Notably, none of the included participants reported a worsening of symptoms or requested to be signposted to the Student Well-being team for further help. These findings are consistent with existing RCTs indicating that digital interventions are safe to use among a diverse user population [74-77]. Furthermore, these findings have implications for future trials emphasizing the importance of such embedded strategies for the successful implementation of digital interventions in diverse settings [78,79].

### Feasibility

Adherence to the Cerina app in this study was comparable to the existing literature on digital CBT-based interventions and mental health apps [67,69-81]. On average, 75% (59/79) of intervention group participants followed the sessions, while 25% (20/79) did not. This finding is consistent with a recent meta-analysis indicating that the attrition rate of mental health apps was 24.1% in the short-term and 35.5% in the longer-term follow-up [24].

The study also provided evidence of attitudinal adherence (emotional investment) to the Cerina app among the participants. In the intervention group, 61% (n=36) of participants followed the core psychoeducational and anxiety-related therapeutic content, while 25% (n=15) accessed the psychoeducational content only, and just a few (n=8, 14%) did not engage with any content. In comparison, in an open feasibility trial with university students in America, just a small portion of the sample (31/117, 26.5%) downloaded a mental health app, and 24% (28/117) of participants used the app only once [32]. In the same study, depression and anxiety-related content of the mental health app were only accessed by 11.1% (13/117) of participants, and users mostly accessed the psychoeducation lessons (27/117, 23.1%) within the app [32]. Thus, it could be argued that engagement features may need to be refined or combined with minimal human support to sustain behavioral adherence, particularly in unsupervised settings.

Several factors may account for the differences observed between this study and the previous feasibility trial by Lattie et al [32]. One explanation could be the differences in the baseline characteristics of the students in both studies. In our sample, the included participants had moderate depression, generalized anxiety, worry, and severe functional impairment, whereas the clinical symptom severity at the baseline is not known for the sample of students in Lattie et al’s [32] study. It is plausible that the higher level of clinical need in our sample increased the perceived relevance of the intervention, thereby enhancing user engagement [79-81]. Alternatively, differences in the nature of the interventions may also explain the variation in adherence.

It is noted that in this study, several engagement features were incorporated into the UI of the Cerina app to optimize the user engagement [36]. It is possible these features improved initial engagement but did not sustain use over time. Future versions of the app might incorporate adaptive elements, social reinforcement features, or real-time feedback to maintain interest. As such, improving sustained user engagement, rather than recruitment, should be the primary focus of refinement. It is also possible that the engagement features helped make the psychoeducational and anxiety-related content more interactive in line with the supportive evidence in the existing literature [38,41]. It may also be beneficial to explore how motivational or cognitive framing in early sessions might better retain users, particularly for worry-specific modules, which showed marginal effects in ITT but stronger effects among completers.

Although the attitudinal adherence among waitlist group participants was similar to the intervention group participants, the behavioral adherence was much lower. It is well established that participation in waitlist groups often heightens expectations about the intervention, which may in turn motivate participants to remain in the study to access the intervention [82]. This pattern was also observed in this study, where significantly more participants in the intervention group (n=41, 71%) failed to complete assessments at t1 and t2 compared to the waitlist control group (n=17, 29%). However, it could be that participants in the waitlist control group got better over time, and they did not feel the need to follow the Cerina app by the time they were given access. Such human-related factors, such as losing motivation, feeling better over time, and not feeling the need to access the intervention, are reported as common reasons for disengagement among waitlist group participants in the existing literature [82].

Despite the attitudinal adherence to the core clinical content of the Cerina app in this study, only a few participants in both the intervention (n=7, 12%) and the waitlist control group (n=1, 4%) completed the desired number of sessions, accessing the whole therapeutic content. This raises interpretive caution, particularly in the completer analyses, which may overestimate the impact of the intervention due to selective dropout. In contrast, the ITT analyses, although more conservative, offer a more comprehensive view of intervention effectiveness across the full sample. Future trials should aim to balance these two approaches and consider hybrid analytic models (eg, multiple imputation or pattern-mixture models) to address bias.

Strategies such as adaptive messaging or brief check-ins from human guides may also reduce dropout and strengthen trial integrity. The low engagement with the whole therapeutic content may point to potential usability issues in the real-world implementation of the Cerina app, despite overall acceptable usability scores. Such challenges are common to digital CBT-based interventions and mental health apps, often leading to poor adoption, implementation barriers, and poor long-term maintenance [79,80]. Known common human-related barriers include disappointment with the lack of human contact, the expectation that the intervention would mimic face-to-face psychotherapy, busy schedules, differing support needs, forgetfulness, and privacy concerns [33,34]. Intervention-related factors include lack of guidance regarding the customized content of the intervention and technical problems (eg, problems with navigation, bugs or glitches, and lack of flexibility with the UI) [33,34]. In this study, several engagement features were used, such as customized notifications, more flexibility regarding navigation, regular check-ins to rate mood, and a chatbot initiating more interaction with the user [36]. However, it could be argued that the evidence base for the effectiveness of these features on long-term adherence and adoption of the digital CBT-based interventions remains limited [38-40,83].

Although the recruitment channels used in this were effective in generating initial interest, the content engagement features alone might not be sufficient to optimize user engagement in the long run. Recent advancements in large language models (LLMs) have provided a promising framework for enhancing engagement features in digital interventions [84]. However, several challenges remain that limit their potential for improving user engagement, such as risk of biases, literacy gaps, and inequalities in data presentation [84]. Additionally, although chatbots and web-based therapist features in particular promise to initiate more interaction with users, the conversation flow can still be perceived as static and redundant by the users [83,84]. It could be that these key challenges contributed to the poor long-term adherence in this study. The ongoing process evaluation is expected to shed more light on these issues, potentially restricting the implementation of the Cerina app in real-life settings, especially after the 2nd week on the app [55]. Future trials could test combinations of interactive components, early engagement tactics, and optional human support to reduce attrition. Importantly, mental health apps using these advanced engagement features should be grounded in a sociocultural-technical framework [84]. Such a framework should provide a global repository of health information to train LLMs to ensure inclusivity, minimize biases, and inequalities in data presentation [84]. Additionally, the sociotechnical limitations of LLMs, such as the structure of a conversation flow with users in mental health settings, should be coproduced with users with lived experience, mental health professionals, and computer scientists [83,84].

### Intervention Effects

Overall, there was an indication that the intervention group achieved better secondary outcomes compared to the waitlist control group. Both completers and ITT analyses showed significant improvements in GAD symptoms from t0 to t2. Therefore, it is pertinent to compare with other similar RCTs investigating the effectiveness of digital CBT-based interventions among clinical [74-77] and student populations [26-31]. The findings of this study align with the recent network meta-analysis supporting both acute and long-term effectiveness of CBT in reducing GAD symptoms among clinical populations [19]. However, the present results contradict the previous meta-analysis, indicating no evidence of a difference in the effects of the digital interventions compared to controls for college students with anxiety symptoms [85]. Similarly, the present findings on GAD symptoms contrast with a previous trial on a transdiagnostic CBT-based digital intervention for university students that found no evidence for the effects of the intervention on anxiety symptoms [26]. Possible explanations of this discrepancy include differences in the comparison group, baseline symptom severity, type of CBT intervention, length, and type of support [26].

Interestingly, although the completers analyses indicated significant improvements in worry symptoms in favor of the intervention with a large effect size, the ITT analyses showed marginally nonsignificant group differences in worry symptoms favoring the intervention. This may be due to early disengagement before the worry-specific modules were completed, suggesting that worry-focused content could be introduced earlier in the program to maximize impact across the full sample. In this study, only a small number of participants completed the desired number of sessions. It is well known that “worry” represents the cognitive part of anxiety that is characterized by unhelpful thoughts such as “I am worthless” and “I am a failure” [46,86]. Since the Cerina app follows a CBT-based protocol, the first 2 sessions focus on psychoeducation on GAD, conceptualizing one’s anxiety symptoms, and setting up goals. Users start working on their worries after the 2nd session [46]. Therefore, though completing the first 2 sessions might have been sufficient to reduce the GAD symptoms in this study, it could be that receiving the intended dose of the intervention is crucial for the treatment of the cognitive aspect of anxiety. Alternatively, the completers were more motivated to use the Cerina app, and this could have inflated their results [87]. A meta-analysis on existing RCTs supports the former argument and suggests that longer treatment (in weeks) was associated with better worry outcomes [73]. To enhance outcomes related to worry, future versions of the intervention could be adapted to include an earlier focus on cognitive aspects of anxiety. Additionally, engagement features could be leveraged to customize the UI to those presenting more severe worry symptoms. They could, for instance, receive notifications and reminders directing them to the sessions and CBT techniques focusing on cognitive aspects of anxiety more often. The effectiveness of these strategies in improving worry outcomes should be tested in future studies.

Despite the well-known close ties between anxiety, depression, and functional impairment (ie, subjective interpretation of the clinical symptoms) [88], the positive trend observed in treating depression did not persist in the ITT analyses. Similarly, there was no favorable effect of the intervention in treating the functional effects of GAD symptoms in the ITT analyses.

With regard to depression symptoms, the present findings contradict the previous meta-analysis, suggesting a large overall effect (*g*=–0.70) of digital CBT-based interventions for GAD on depression symptoms [73]. One plausible explanation for these contradictory results could be related to differences in the types of CBT-based interventions between the current and former studies. This study used a targeted CBT-based intervention for treating a single condition (GAD symptoms) rather than a transdiagnostic (unified) CBT-based intervention, which addresses overlapping characteristics of GAD and depression, such as uncontrollable worry about multiple future events and rumination (ie, repeatedly thinking about past or current concerns) [26]. Therefore, it could be argued that completing more sessions focusing on understanding and managing worry among the completers potentially had an effect on reducing their depression symptoms in this study.

Regarding functional impairment, the discrepancy between the ITT and completers analyses could be explained by greater engagement with the intervention among completers. Consistent with the present findings, a previous meta-analysis reported a large overall effect of digital CBT-based interventions on functional outcomes (*g*=−0.66) [73]. This suggests that user engagement and completing the intended number of sessions (ie, receiving the intended dose of the intervention) could play an important role in improving the functional effects of anxiety [86].

It is also likely that the improvements in secondary outcomes can be attributed to the group status (intervention group) rather than the access to usual care, which cannot be ruled out in this study. Even though receiving psychological support at baseline showed a slight trend toward improving secondary outcomes, all support measures (ie, professional support, psychological support from the informal network, and helpfulness of the professional support) generally showed weak correlations with the improvements in secondary outcomes. This could indicate that accessing usual care (ie, psychological support from informal networks or professional support) alone may not be a strong predictor of improvements in secondary outcomes in this study.

### Clinical Implications

This pilot feasibility trial highlights several important clinical implications. Findings suggest that digital CBT interventions such as Cerina can be feasibly and safely integrated into university well-being services, offering accessible and scalable support for student mental health. These tools are particularly valuable for students who may be reluctant to seek face-to-face help due to stigma, limited time, or other barriers.

Universities could use this emerging evidence to inform the development, procurement, or commissioning of digital mental health resources. Specific features likely to enhance uptake and sustained engagement include user-friendly onboarding, options for personalization, and interactive content that maintains interest over time.

Digital CBT interventions can also be embedded within stepped care models in both university and primary care settings. As low-intensity, first-line options, platforms like Cerina may help manage the growing demand for mental health support by providing early intervention and reducing pressure on mental health services and general practices. In primary care, these tools could assist in more effective triage, support self-management, and facilitate continuity of care for people with emerging or mild symptoms, or those referred but awaiting treatment by mental health services.

Importantly, presenting digital CBT as a universal well-being resource rather than a treatment may help reduce stigma and increase engagement. As both education and health systems look to expand capacity and address mental health needs more efficiently, interventions like Cerina can offer a flexible, cost-effective, and user-centered solution.

### Strengths and Limitations

There are several limitations to this study. As discussed above, the attrition rate was one of the main limitations, as this can result in an imbalance occurring by chance and can introduce bias [87]. However, our analyses confirmed that the data missing due to attrition were missing completely at random, and variables in this study were unaffected by the attrition between time points. Notably, completer and ITT analyses showed differing outcomes. While ITT offers a more conservative estimate of effectiveness, completers analyses may be biased due to dropout. [87]. It could be that many participants dropped out due to the inefficacy of the intervention, or they could have felt better and did not feel the need to use the Cerina app anymore. Therefore, the completers analyses might fail to capture the limitations or potential downsides of the intervention [87]. Understanding patterns of dropout can inform future designs aimed at minimizing attrition, such as adaptive intervention strategies or just-in-time support.

Another limitation relates to the sample size. Since the sample size is attenuated in a completers analysis, the statistical power is reduced. Additionally, the benefits of randomization may be compromised due to changes in group composition [87]. In contrast, the ITT sample in the present study comprises all participants who were randomized, reducing the likelihood of selection bias. Additionally, to minimize any bias in the ITT sample, we tested several imputation models and selected a linear regression model, which confirmed the data distribution while preserving an acceptable level of variance within the imputed data.

Additionally, this study was designed as a feasibility pilot RCT; therefore, it was not powered to detect significant intervention effects [36]. Despite this limitation, we exceeded the intended sample size, and both the completers and the ITT analyses indicated significant group differences in GAD symptoms in favor of the intervention group with a medium effect size. This finding shows the meaningful impact of the intervention and suggests that the effect size of the intervention is likely to be bigger in a future full-scale RCT.

Another important limitation of this study concerns the operationalization of adherence (referred to as engagement in the Staudt model) [37,48]. The way we defined behavioral and attitudinal adherence might not be representative of the multidimensionality of the construct. For example, the way users navigate sessions or engagement sequences was not measured in this study, and they may yield additional insights [34,47]. Furthermore, unlike the approach taken by Hanano et al [48], we did not operationalize attitudinal adherence as “the extensiveness of users’ open-ended reflective responses on weekly check-ins” [47]. Instead, we focused on participants’ engagement with the content and relied on the flow of the CBT protocol to define different levels of attitudinal adherence in this study [36]. This approach is more efficient and clinically relevant [66]. Another approach is to compare the qualitative and quantitative analyses of user engagement [55]. The process evaluation will enable us to combine different aspects and facilitate a more accurate understanding of adherence in this study [55].

A further limitation is that the sample of this study is not representative of the university student population in Northern Ireland, which limits the generalizability of the findings [36]. However, the demographic and clinical characteristics of the current sample mimic the previous findings on these characteristics among student populations in Northern Ireland [42,70] and globally [88].

Finally, it is well established that participation in a waitlist control group can increase motivation to remain in the trial and may elevate participants’ expectations regarding the intervention’s effectiveness [89,90]. This cannot be ruled out in this study. The waitlist control group was chosen as a comparison group to offer the possibility of completing the intervention to a vulnerable population who might not have access to any form of psychological help otherwise.

There are several strengths of this study that warrant acknowledgment. First, a recent audit of the health apps available in the Apple store highlighted that less than half (n=220, 44%) claimed to be evidence-based, and more than 70% of these evidence-based apps relied on low-quality evidence [91]. Moreover, significant methodological limitations were identified in the small subset of developers who published studies [91]. This pilot feasibility study addresses this gap and contributes high-quality evidence on the safety, feasibility, and effectiveness of mental health apps in general.

Second, the low-threshold CBT-based mobile app in this study was tailored to the student population, developed using co-design principles [36]. This approach informed the prioritization of specific engagement features and enabled the evaluation of their impact on user engagement. The study contributes to the growing body of evidence suggesting that further research, particularly with longer-term follow-up, is needed to better understand the role of engagement features in treatment outcomes [38,44].

Another notable strength is the collaborative partnership underpinning the study involving Ulster University, the Ulster University Student Well-being team, and Cerina Health (a mental health start-up company). This collaboration contributed to both the reach and acceptability of the Cerina app among students, supporting its alignment with the goals of university-based digital mental health interventions.

### Conclusions

In conclusion, the design of this trial provides the groundwork for future development of full-scale RCTs embedded in Student Well-being services. Though this study was designed as a feasibility pilot trial, it exceeded the intended sample size (n=90) and was successfully conducted in a university setting. Furthermore, the results highlight the likely effects of the Cerina app in reducing anxiety symptoms, offering a basis for further research and potential implementation of the intervention in broader contexts to alleviate anxiety. Importantly, the usability issues identified in this study underscore the need for iterative refinement of digital CBT-based interventions. Ongoing feedback and the evaluation of long-term effects of engagement features will be essential to enhancing user experience and sustaining engagement in future trials.

## Appendix

Multimedia Appendix 1 CONSORT 2010 checklist.

Multimedia Appendix 2 CONSORT-eHEALTH checklist (V 1.6.1).

Multimedia Appendix 3 Baseline characteristics.

Multimedia Appendix 4 (Left) the change in GAD7 score distributions across t0-t2, (middle) the datapoint swam of GAD7 total scores for t0-t2, (right) the change in mean GAD7 score across t0-t2 among completers.

Multimedia Appendix 5 (Left) the change in PSWQ score distributions across t0-t2, (middle) the datapoint swam of GAD7 total scores for t0-t2, (right) the change in mean PSWQ score across t0-t2 among completers.

Multimedia Appendix 6 (Left) the change in PHQ9 score distributions across t0-t2, (middle) the datapoint swam of GAD7 total scores for t0-t2, (right) the change in mean PHQ9 score across t0-t2 among completers.

Multimedia Appendix 7 (Left) the change in WSAS score distributions across t0-t2, (middle) the datapoint swam of GAD7 total scores for t0-t2, (right) the change in mean WSAS score across t0-t2 among completers.

Multimedia Appendix 8 (Left) the change in GAD7 score distributions across t0-t2, (middle) the datapoint swam of GAD7 total scores for t0-t2, (right) the change in mean GAD7 score across t0-t2 among the ITT sample.

Multimedia Appendix 9 (Left) the change in PSWQ score distributions across t0-t2, (middle) the datapoint swam of GAD7 total scores for t0-t2, (right) the change in mean PSWQ score across t0-t2 among the ITT sample.

Multimedia Appendix 10 (Left) the change in WSAS score distributions across t0-t2, (middle) the datapoint swam of GAD7 total scores for t0-t2, (right) the change in mean WSAS score across t0-t2 among the ITT sample.

Multimedia Appendix 11 (Left) the change in PHQ9 score distributions across t0-t2, (middle) the datapoint swam of GAD7 total scores for t0-t2, (right) the change in mean PHQ9 score across t0-t2 among the ITT sample.

Multimedia Appendix 12 Correlation Matrix.

## Abbreviations

CBT: cognitive behavioral therapy
CMD: common mental disorder
CONSORT: Consolidated Standards of Reporting Trials
CONSORT-EHEALTH: Consolidated Standards of Reporting Trials of Electronic and Mobile Health Applications and Online Telehealth
GAD: generalized anxiety disorder
GAD-7: Generalized Anxiety Disorder Scale-7
GP: general practitioner
ITT: intention to treat
LLM: large language model
PHQ-9: Patient Health Questionnaire-9
PSWQ: Penn State Worry Questionnaire
RCT: randomized controlled trial
SUS: System Usability Scale
UI: user interface
WHO: World Health Organization
WSAS: Work Social Adjustment Scale
