# Supplementary material for: Cerina—Cognitive Behavioral Therapy–Based Mobile App for Managing Generalized Anxiety Disorder Symptoms Among University Students: Results From a Pilot Feasibility Randomized Controlled Trial
Source: JMIR Mhealth Uhealth. 2025 Oct 9;13:e70691. doi: 10.2196/70691 (PMC12550456; doi:10.2196/70691)
Supplement: Multimedia Appendix 3 [file mhealth_v13i1e70691_app3.docx]

| Characteristics | | Intervention Participants  (*N*=79) | | Waitlist-Control Participants  (*N*=79) | | Total  (*N*=158) | | *P* Value^a^ | |
| --- | --- | --- | --- | --- | --- | --- | --- | --- | --- |
| **Age (N, %)** |  | | | |  |  | | .13 | |
| 18-30 | 52 (65.8%) | | | | 52 (65.8%) | 104 (65.8%) | |  | |
| 30-45 | 26 (32.9%) | | | | 21 (26.6%) | 47 (29.7%) | |  | |
| 45+ | 1 (1.3%) | | | | 6 (7.6%) | 7 (4.4%) | |  | |
| **Gender (N,%)** |  | | | |  |  | | .41 | |
| Male | 16 (20.3%) | | | | 12 (15.2%) | 28 (17.7%) | |  | |
| Female | 62 (78.5%) | | | | 67 (84.8%) | 129 (81.6%) | |  | |
| Other | 1 (1.13%) | | | | 0 | 1 (0.6%) | |  | |
| **Education Level (N,%)** |  | | | |  |  | | .24 | |
| GCSE | 1 (1.3%) | | | | 1 (1.3%) | 2 (1.3%) | |  | |
| A level | 17 (21.5%) | | | | 17 (21.5%) | 34 (21.5%) | |  | |
| Undergraduate | 45 (57%) | | | | 33 (41.8%) | 78 (49.4%) | |  | |
| Postgraduate | 14 (17.7%) | | | | 26 (32.9%) | 40 (25.3%) | |  | |
| Other | 2 (2.5%) | | | | 2 (2.5%) | 4 (2.5%) | |  | |
| **Support Network (N,%)^b^** |  | | | |  |  | | .12 | |
| Friends | 4 (5.1%) | | | | 7 (8.9%) | 11 (7%) | |  | |
| Family | 6 (7.6%) | | | | 9 (11.45) | 15 (9.5%) | |  | |
| Partner | 11 (13.9%) | | | | 8 (10.1%) | 19 (12%) | |  | |
| Other | 7 (8.9%) | | | | 6 (7.6%) | 13 (8.2%) | |  | |
| Friends, family | 22 (27.8%) | | | | 20 (25.3%) | 42 (26.5%) | |  | |
| Family, partner | 4 (5.1%) | | | | 9 (11.4%) | 13 (8.2%) | |  | |
| Friends, partner | 6 (7.6%) | | | | 6 (7.6%) | 12 (7.6%) | |  | |
| Friends, family, partner | 3 (3.8%) | | | | 9 (11.4%) | 12 (7.6%) | |  | |
| All of the above | 16 (20.3%) | | | | 5 (6.3%) | 21 (13.3%) | |  | |
| **Professional Support^b^** **(N,%)** |  | | | |  |  | | .30 | |
| No | 40 (50.6%) | | | | 43 (54.4%) | 83 (52.5%) | |  | |
| Yes | 1 (1.3%) | | | | 5 (6.3%) | 6 (3.8%) | |  | |
| GP | 11 (13.9%) | | | | 10 (12.7%) | 21 (13.3%) | |  | |
| Psychologist | 2 (2.5%) | | | | 0 | 2 (1.3%) | |  | |
| Student Wellbeing Team | 7 (8.9%) | | | | 4 (5.1%) | 11 (7%) | |  | |
| Psychiatrist | 1 (1.3%) | | | | 1 (1.3%) | 2 (1.3%) | |  | |
| Counsellor | 7 (8.9%) | | | | 4 (5.1%) | 11 (7%) | |  | |
| Psychologist, Psychiatrist | 1 (1.3%) | | | | 0 | 1 (0.6%) | |  | |
| GP, Student Wellbeing Team | 2 (2.5%) | | | | 1 (1.3%) | 3 (1.9%) | |  | |
| Psychiatrist, Student Wellbeing Team | 1 (1.3%) | | | | 0 | 1 (0.6%) | |  | |
| GP, Student Wellbeing Team, Counsellor | 3 (3.8%) | | | | 0 | 3 (1.9%) | |  | |
| Other | 3 (3.8%) | | | | 7 (8.9%) | | 10 (6.3%) | |  |
| **Helpfulness of the psychological support (N,%)** | | |  | |  | |  | | .40 |
| Unhelpful | | | 4 (5.1%) | | 1 (1.3%) | | 5 (3.2%) | |  |
| Neither helpful nor unhelpful | | | 51 (64.6%) | | 54 (68.6%) | | 105 (66.5%) | |  |
| Helpful | | | 20 (25.3%) | | 17 (21.5%) | | 37 (23.4%) | |  |
| Extremely helpful | | | 4 (5.1%) | | 7 (8.9%) | | 11 (7%) | |  |
| **Medication Status (N,%)** | | |  | |  | |  | | .59 |
| No | | | 50 (63.3%) | | 55 (69.6%) | | 105 (66.5%) | |  |
| Yes, I have started recently | | | 2 (2.5%) | | 2 (2.5%) | | 4 (2.5%) | |  |
| Yes, on a stable dose for the past 2 months | | | 4 (5.1%) | | 6 (7.6%) | | 10 (6.3%) | |  |
| Yes, on a stable dose for the past 6 months | | | 23 (29.1%) | | 16 (20.3%) | | 39 (24.7%) | |  |
| Generalised Anxiety (GAD7, M,SD) | | | 12.25, 3.70 | | 11.03, 3.89 | | 11.64, 3.84 | | .23 |
| Depression (PHQ9, M,SD) | | | 13.73, 5.16 | | 11.68, 4.99 | | 12.71, 5.16 | | .26 |
| Worry (PSWQ-PW, M,SD) | | | 50.96, 8.65 | | 51.19, 8.90 | | 51.08, 8.75 | | .09 |
| Work Social Adjustment (WSAS, M,SD) | | | 20.94, 7.85 | | 17.89, 8.61 | | 19.41, 8.53 | | .39 |

^a^ P values are based on t-test or chi-square test

^b^ Participants were able to choose more than one option
