# Supplementary figures and images for: Cerina—Cognitive Behavioral Therapy–Based Mobile App for Managing Generalized Anxiety Disorder Symptoms Among University Students: Results From a Pilot Feasibility Randomized Controlled Trial
Source: JMIR Mhealth Uhealth. 2025 Oct 9;13:e70691. doi: 10.2196/70691 (PMC12550456; doi:10.2196/70691)

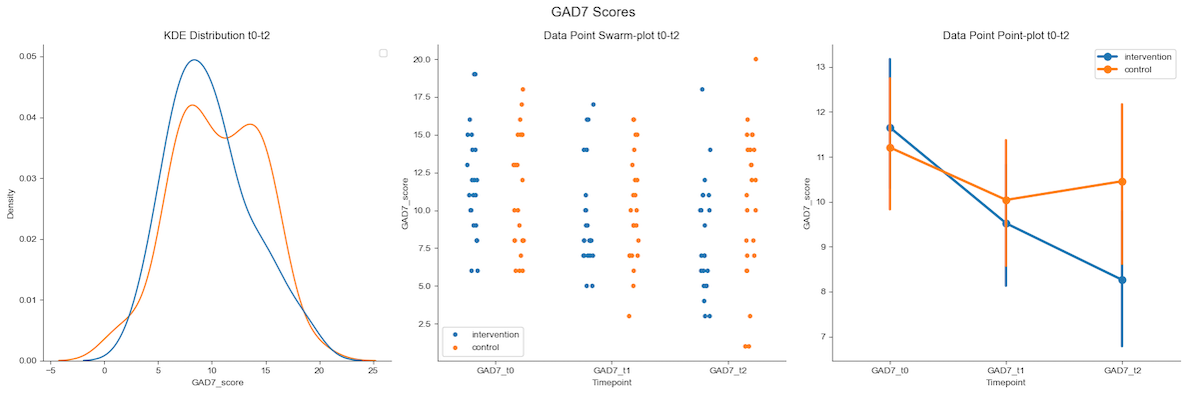

Supplement: Multimedia Appendix 4 [file mhealth_v13i1e70691_app4.png]

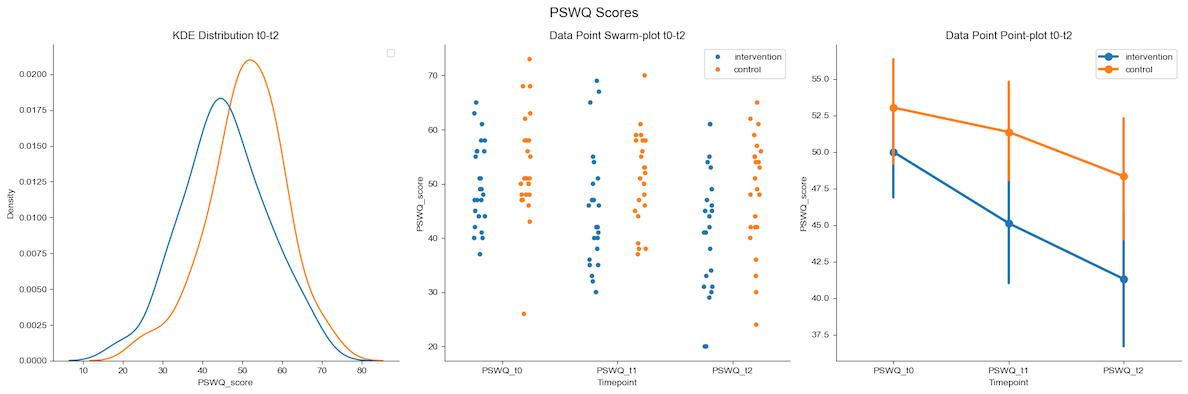

Supplement: Multimedia Appendix 5 [file mhealth_v13i1e70691_app5.png]

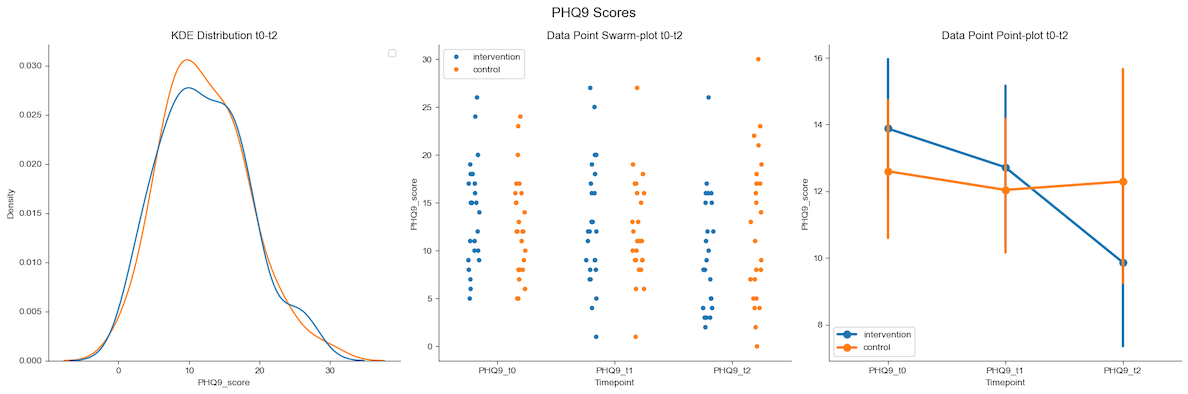

Supplement: Multimedia Appendix 6 [file mhealth_v13i1e70691_app6.png]

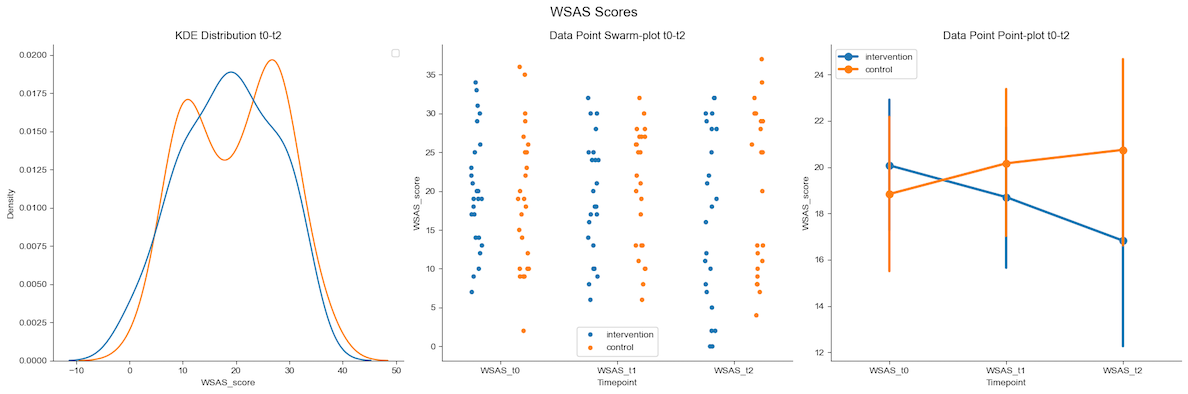

Supplement: Multimedia Appendix 7 [file mhealth_v13i1e70691_app7.png]

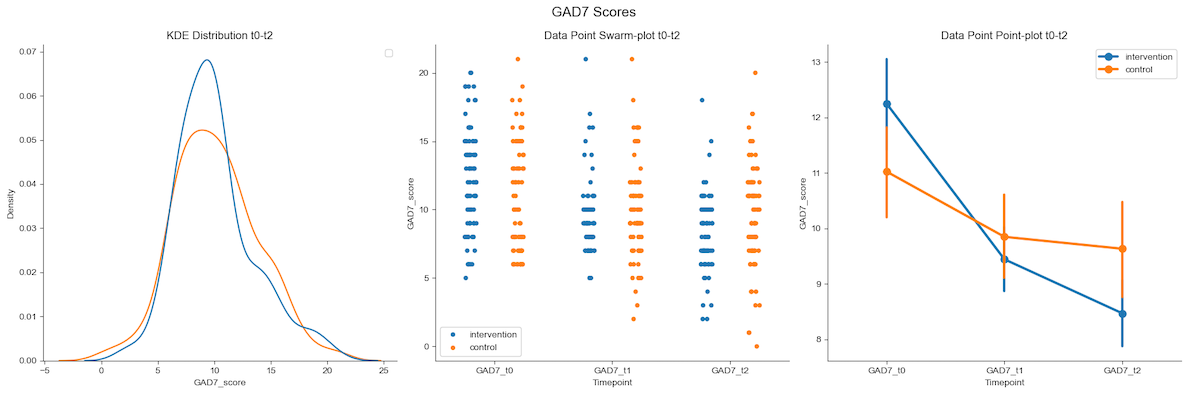

Supplement: Multimedia Appendix 8 [file mhealth_v13i1e70691_app8.png]

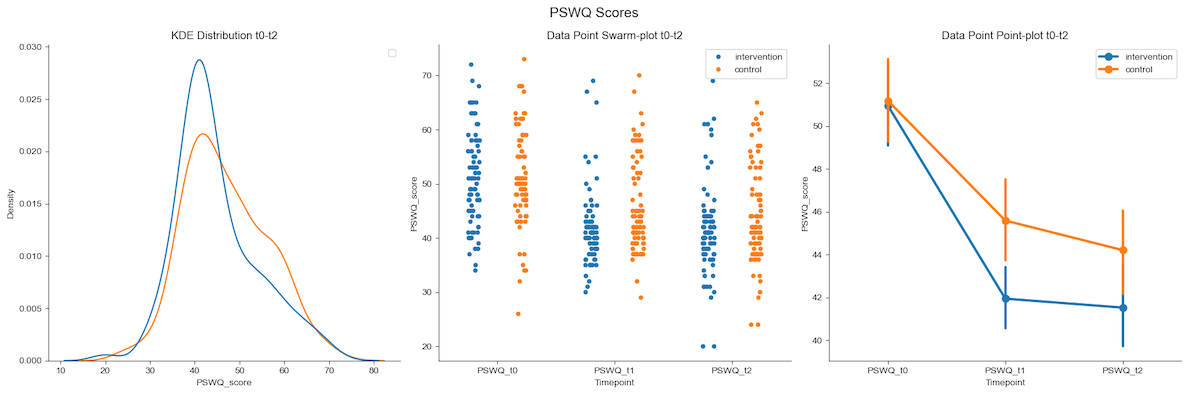

Supplement: Multimedia Appendix 9 [file mhealth_v13i1e70691_app9.png]

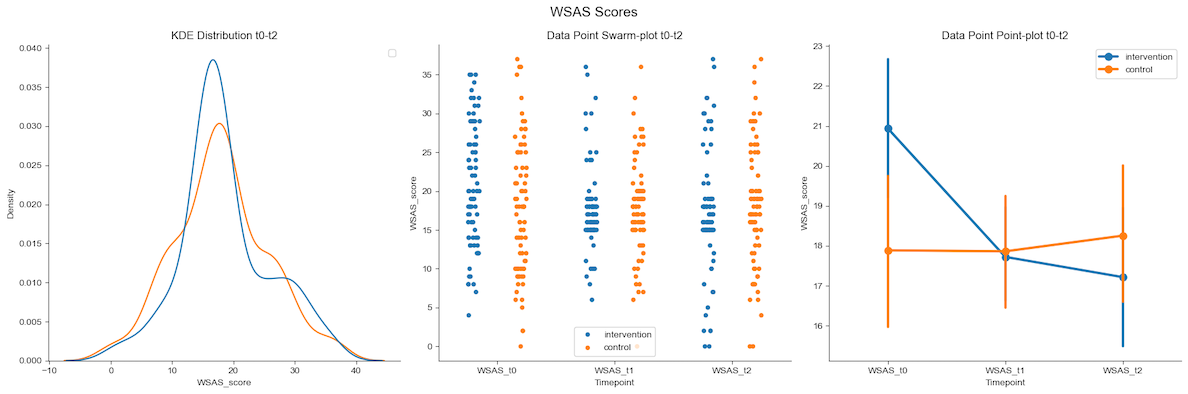

Supplement: Multimedia Appendix 10 [file mhealth_v13i1e70691_app10.png]

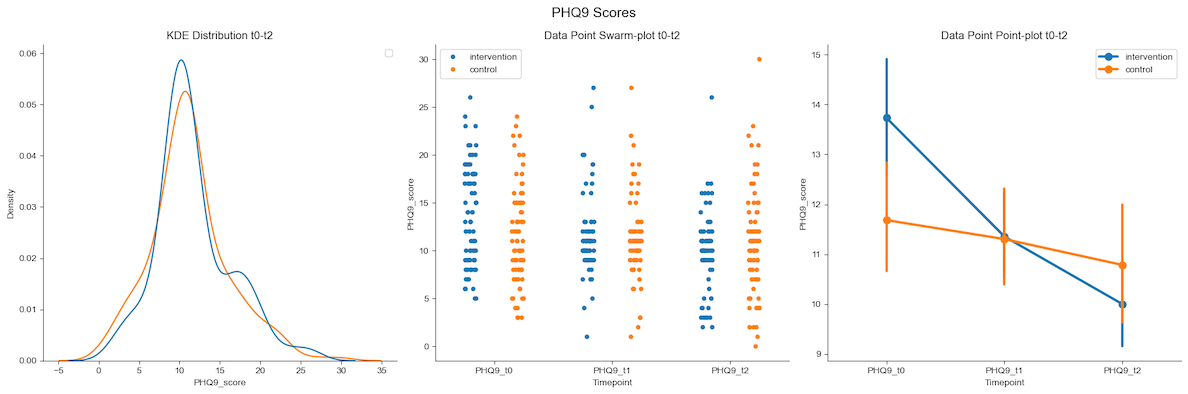

Supplement: Multimedia Appendix 11 [file mhealth_v13i1e70691_app11.png]

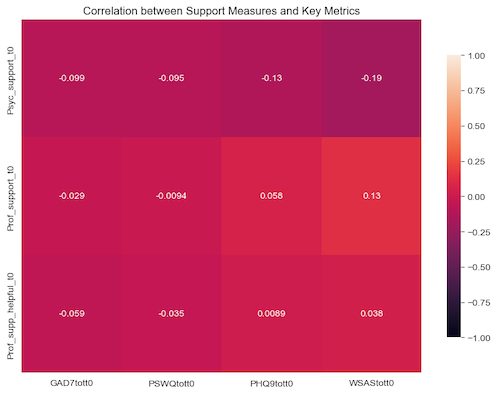

Supplement: Multimedia Appendix 12 [file mhealth_v13i1e70691_app12.png]
